# Factors Influencing Maternal Readiness for Hospital Discharge: A Cross‐Sectional Study

**DOI:** 10.1002/nop2.70562

**Published:** 2026-04-20

**Authors:** Zhijing Feng, Xiaofei Tian, Haiyang Zheng, Jiaxin Li, Jingwen Ye, Piaopiao Ma, Guimei Yao, Miaomiao Qiu, Xinxin Li

**Affiliations:** ^1^ Department of Gynecology and Obstetrics The Seventh Affiliated Hospital of Sun Yat‐Sen University Shenzhen China; ^2^ Nursing Department The Seventh Affiliated Hospital of Sun Yat‐Sen University Shenzhen China

**Keywords:** health literacy, influencing factors, maternal readiness for hospital discharge, nursing, postpartum period, quality of discharge teaching

## Abstract

**Aim:**

To assess mainland Chinese mothers' readiness for hospital discharge and related factors after childbirth.

**Design:**

A descriptive cross‐sectional study.

**Methods:**

Convenience sampling was employed to select women who gave birth at the Department of Obstetrics and Gynaecology of a tertiary hospital in Shenzhen, China, between May 2024 and February 2025. Maternal readiness for hospital discharge was evaluated using the Chinese version of the Readiness for Hospital Discharge Study‐New Mother Form. Data were analysed using an independent sample *t*‐test, one‐way analysis of variance, Kruskal–Wallis test, Spearman correlation and multiple linear regression analysis.

**Results:**

The study included 308 mothers aged 20–41 years (31.15 ± 4.14). The total readiness score for hospital discharge was in the range of 54–180 (132.17 ± 22.59). The ‘delivery’ dimension in the Quality of Discharge Teaching Scale‐New Mother Form and the ‘lifestyle and behavior’ and ‘basic skills’ dimensions in the Perinatal Maternal Health Literacy Scale were independent factors influencing readiness for hospital discharge (*p* < 0.05) among mothers after childbirth.

**Conclusions:**

Maternal readiness for hospital discharge must be enhanced. This could be done by improving the quality of discharge teaching and maternal health literacy.

**Patient or Public Contribution:**

No patient or public contribution.

## 1 Introduction

International focus on maternal health has been centred on reducing maternal mortality; however, many postpartum complications that seriously affect women's health and well‐being have been neglected (Vogel et al. 2024). Simultaneously, with the continued development of rapid postpartum rehabilitation, maternal hospital stays are significantly shorter (Caughey et al. 2018), which may result in inadequate postpartum health education and lower levels of readiness for hospital discharge (RHD) (Harvey et al. 2023). Lower levels of RHD increase the incidence of puerperal complications, neonatal illnesses and postpartum readmissions, which significantly affect the quality of postpartum life and raise healthcare costs and resource use even further (Smith et al. 2022). Therefore, it is crucial to assess maternal RHD (Moradi et al. 2018).

According to the recommendations for maternal and infant postpartum healthcare developed by the World Health Organization (WHO) (Wojcieszek et al. 2023), RHD includes the physiological stability of the mother and infant, the mother's knowledge and competence in self‐care and infant care, adequate social support and access to obstetric healthcare resources. Assessing RHD can help accurately determine when a patient is ready for discharge and better predict potential challenges that may arise after discharge; hence, it is considered a reliable indicator of maternal health outcomes (Lau et al. 2023).

Factors such as the quality of discharge teaching, vaginal delivery, multiple children, feeding mode, social support, family functioning and parenting ability are closely linked to maternal RHD (Smith et al. 2022; Malagon‐Maldonado et al. 2017). However, data from mainland China on this topic are limited and inconsistent. Therefore, this study aimed to assess maternal RHD levels, identify deficiencies in predischarge preparation and explore the influencing factors, with the further aim of providing a theoretical foundation for the development of a clinical intervention program to improve maternal RHD.

## 2 Materials and Methods

### 2.1 Study Design

This was a descriptive cross‐sectional study.

### 2.2 Participants

Convenience sampling was employed to select women who gave birth at the Department of Obstetrics and Gynaecology of a tertiary hospital in Shenzhen, China, between May 2024 and February 2025. The inclusion criteria were as follows: (1) individuals discharged on the same day based on medical advice; (2) those who were conscious, able to communicate verbally and capable of completing the questionnaire; and (3) those who provided informed consent and voluntarily agreed to participate. The exclusion criteria were as follows: (1) individuals with cognitive or communication disorders and (2) those with physical limitations that hindered engagement with the study.

### 2.3 Data Collection

Two nurses with more than 5 years of experience in the postpartum ward were selected and trained to collect data (Xia et al. 2023). On the day of discharge, the investigator provided eligible mothers with a standardised explanation of the purpose of the survey. Those who agreed to participate were asked to sign an informed consent form and complete a questionnaire, which took approximately 20 min. The investigator collected and reviewed the quality of the questionnaires and asked the participants to complete their responses immediately if there were any missing items. Subsequently, two researchers with master's degrees re‐evaluated the collected data during the data entry process. Moreover, questionnaires displaying significant patterns (same options or options in a loop) in the responses were excluded.

### 2.4 Measures

#### 2.4.1 Measurement Instrument for the Outcome Variable

RHD was evaluated using the Chinese version of the Readiness for Hospital Discharge Study‐New Mother Form (RHDS‐NMF). This scale was originally developed by Weiss et al. (2006) and translated into Chinese by Xing et al. (2020). It comprises 19 items categorised into four dimensions: personal status (the physical‐emotional state of the patient immediately before discharge, 4 items); knowledge (the perceived adequacy of information needed to respond to common concerns and problems in the post‐hospitalisation period, 7 items); coping ability (the perceived ability of the patient to self‐manage personal and healthcare needs after discharge, 3 items); and expected social support (emotional and instrumental assistance expected to be available following hospital discharge, 4 items). The first item is a yes/no question and is not included in the total score. The remaining items are scored on a scale from 0 to 10, where ‘0’ indicates ‘completely unknown’ or ‘not at all’, while ‘10’ denotes ‘completely known’ or ‘completely able’. Patients selected scores that reflected their circumstances. The sum of all item scores determined the scale's total score, which ranged from 0 to 180. Higher scores indicated greater RHD. Cronbach's alpha coefficient for the original scale used in postpartum mothers was 0.838 (Weiss et al. 2006; Weiss and Piacentine 2006), while the Chinese version of the scale demonstrated a Cronbach's alpha coefficient of 0.901 (Xing et al. 2020). In this study, the coefficient for the scale was 0.802.

#### 2.4.2 Measurement Instruments for the Study Variables

The WHO (Wojcieszek et al. 2023) highlights that maternal RHD encompasses the physiological stability of the mother and infant, the mother's knowledge and competence in self‐care and infant care, adequate social support and access to obstetric healthcare resources, while Smith et al. (2022) and Malagon‐Maldonado et al. (2017) indicated that the quality of discharge teaching, vaginal delivery, having multiple children, feeding mode, social support, family functioning and parenting ability are closely linked to maternal RHD (Smith et al. 2022; Malagon‐Maldonado et al. 2017). Therefore, this study included the following variables:

First, a self‐designed sociodemographic characteristics questionnaire was used to collect data on age, height, weight, body mass index (BMI), education level, ethnicity, marital status, religion, occupation, per capita monthly household income, medical payment, delivery mode, main caregiver and planned feeding.

Second, the quality of discharge teaching (QDT) was measured using the Chinese version of the Quality of Discharge Teaching Scale‐New Mother Form (OB‐QDTS). This scale was initially compiled by Weiss et al. (2007) and translated into Chinese by Wen et al. (2021). It comprises 27 items in 3 dimensions: ‘content needed’—information mothers should receive before discharge (items 1a–7a); ‘content received’—information mothers actually receive before discharge (items 1b–7b); and ‘delivery’—skills and effectiveness of guidance provided by nursing staff (items 8–20). The ‘content needed’ and ‘content received’ responses were assessed in relation to how well they corresponded with each other. Responses were rated on a scale of 0–10, where 0 indicates ‘not at all/none’ and 10 signifies ‘completely/very much’. The total score for the scale was calculated by summing the scores from the ‘content received’ and ‘delivery’ dimensions and dividing by the total number of items. The scoring system classifies QDT into four categories: < 7.0, representing insufficient QDT; 7.0–7.9, representing medium QDT; 8.0–8.9, representing high QDT; and 9.0–10.0, representing extremely high QDT. The original scale had a Cronbach's alpha coefficient of 0.870 (Weiss et al. 2007), whereas the Chinese adaptation had a value of 0.953 (Wen et al. 2021). In this study, Cronbach's alpha coefficient for the scale was 0.821.

Third, health literacy (HL) was assessed using the Perinatal Maternal Health Literacy Scale (PMHLS), which was developed by Dai in Chinese (Dai 2014). The scale comprises 34 items, including three dimensions: basic knowledge and concepts (maternal cognition in relation to the normal physiological conditions of the mother and her newborn postpartum, 15 items); lifestyle and behaviour (maternal behaviours conducive to the mother's health and breastfeeding postpartum, 12 items); and basic skills (maternal skills concerning breastfeeding and neonatal care postpartum, 7 items). An answer of ‘yes’ is assigned a score of 1, while an answer of ‘no’ or ‘don't know’ is assigned a score of 0. The sum of the scores of all items is the total score of the questionnaire; the higher the score, the higher the level of HL. Cronbach's alpha coefficient for this scale was 0.819 (Dai 2014). In this study, Cronbach's alpha coefficient was 0.791.

### 2.5 Sample Size

Multiple linear regression analysis generally requires a sample size of at least 10 times the number of independent variables (Zheng and Yu 2018). This study included 18 independent variables. The sample size was increased by 10% to avoid invalid questionnaire responses, with the final sample of 308 considerably exceeding the minimum of 200 required.

### 2.6 Ethical Consideration

This study was approved by the Hospital Research Ethics Committee where the research was conducted (KY‐2024‐393‐01). The study complied with the provisions of the Declaration of Helsinki, as revised in Edinburgh in 2000. All participants were assured that their data would remain confidential. Participation was entirely voluntary; the participants signed consent forms and could withdraw from the study at any time (Xia et al. 2023).

### 2.7 Data Analysis

SPSS 27.0 software was used for data analysis. The mean, standard deviation, frequency and composition ratio were used to describe the sociodemographic characteristics and variables. Comparisons between two variables were performed using an independent sample *t*‐test (both sets of data were normally distributed). Comparisons between multiple variables were performed using one‐way analysis of variance (all sets of data that met normality and homogeneity of variance criteria) or the Kruskal–Wallis test (any set of data that did not meet normality or homogeneity of variance criteria). Spearman's correlation analysis (any set of data that did not meet normality criteria) was performed to determine the correlation between the RHD, QDT and HL scores. Significant factors were analysed using stepwise multiple linear regression analysis. A two‐sided *p* < 0.05 was considered statistically significant.

## 3 Results

### 3.1 Sociodemographic Characteristics of the Participants

A total of 357 questionnaires were collected in this study, of which 308 were valid (validity rate: 86.27%). The age range of the participants was 20–41 years (31.15 ± 4.14) and their BMI was 16.42–41.41 (24.75 ± 3.41). The other sociodemographic characteristics are shown in Table 1.

**TABLE 1:** Sociodemographic characteristics of the participants and differences in the RHD scores among the variables (*n* = 308).

Characteristics	*n* (%)	RHD ($\bar{X}$ ± *S*)	Statistics	*p*
Age			1.117^a^	0.773
≤ 25	21 (6.8)	128.19 ± 21.23		
26–30	115 (37.3)	131.98 ± 20.98		
31–35	120 (39)	131.40 ± 23.80		
> 35	52 (16.9)	135.94 ± 18.97		
BMI (kg/m^2^)			2.294^b^	0.078
< 18.5	4 (1.3)	127.50 ± 17.14		
18.5–24.9	174 (56.5)	129.44 ± 23.41		
25–29.9	107 (34.7)	136.55 ± 21.11		
≥ 30	23 (7.5)	132.17 ± 22.59		
Education level			0.532^b^	0.661
Junior high school or below	37 (12.0)	135.46 ± 26.50		
High school or technical secondary school	42 (13.6)	130.05 ± 25.54		
Junior college or bachelor's degree	207 (67.2)	131.70 ± 21.62		
Master's degree or above	22 (7.1)	135.05 ± 18.92		
Ethnicity			1.320^c^	0.188
Han ethnicity	284 (92.2)	131.67 ± 22.70		
Minority ethnicity	24 (7.8)	138.00 ± 20.67		
Marital status			0.501^c^	0.617
Unmarried	9 (2.9)	128.44 ± 28.71		
Married	299 (97.1)	132.28 ± 22.43		
Religion			0.686^c^	0.493
Yes	17 (5.5)	135.82 ± 21.93		
No	291 (94.5)	131.95 ± 22.64		
Occupation			0.339^b^	0.713
Employed	216 (70.1)	132.32 ± 22.37		
Individual operation	40 (13.0)	133.95 ± 25.38		
Unemployed	52 (16.9)	130.13 ± 21.49		
Per capita monthly household income			0.142^b^	0.868
< 5000¥	40 (13.0)	133.75 ± 25.34		
5000–10,000¥	140 (45.5)	131.61 ± 22.58		
≥ 10,000¥	128 (41.5)	132.28 ± 21.83		
Medical payment			0.429^a^	0.807
Maternity insurance	288 (93.5)	132.16 ± 22.51		
Other insurance	7 (2.3)	131.29 ± 39.02		
Self‐funded	13 (4.2)	132.77 ± 13.09		
Delivery mode			0.589^c^	0.576
Vaginal delivery	221 (71.8)	131.86 ± 22.39		
Caesarean delivery	87 (28.2)	132.95 ± 23.18		
Main caregiver			0.274^b^	0.844
Parents	42 (13.6)	131.26 ± 18.11		
Parents in law	119 (38.6)	132.98 ± 23.55		
Maternity matron	92 (29.9)	132.84 ± 23.02		
Husband	55 (17.9)	129.96 ± 23.20		
Planned feeding			0.287^a^	0.866
Breast feeding	87 (28.2)	131.77 ± 23.62		
Mixed feeding	193 (62.7)	133.13 ± 20.57		
Bottle	28 (9.1)	126.71 ± 31.21		
Quality of discharge teaching grade			35.968^a^	< 0.001
Insufficient	77 (25.0)	119.88 ± 25.59		
Medium	71 (23.1)	130.35 ± 19.56		
High	90 (29.2)	136.53 ± 19.00		
Very high	70 (22.7)	141.90 ± 20.04		

^a^ Kruskal–Wallis test, *Z*.

^b^ One‐way ANOVA, *F*.

^c^ Independent sample *t*‐test, *t*.

### 3.2 Differences in RHD Scores Among the Variables

The results showed no statistically significant effects in terms of age, BMI, education level, ethnicity, marital status, religion, occupation, per capita monthly household income, medical payment, delivery mode, main caregiver, or planned feeding on maternal RHD. However, the QDT grade demonstrated a statistically significant effect on maternal RHD (Table 1).

### 3.3 RHD, QDT and HL Scores

The RHD score was 54–180 (132.17 ± 22.59), and the sub‐dimension scores were 30.11 ± 4.88 (personal status), 49.78 ± 11.26 (knowledge), 21.38 ± 4.62 (coping ability) and 30.89 ± 5.47 (expected social support) (Table 2).

**TABLE 2:** RHD, QDT and HL scores (*n* = 308).

Variables	Item number	Full score	Actual score
RHD	18	0–180	132.17 ± 22.59
Personal status	4	0–40	30.11 ± 4.88
Knowledge	7	0–70	49.78 ± 11.26
Coping ability	3	0–30	21.38 ± 4.62
Expected social support	4	0–40	30.89 ± 5.47
QDT	27	0–10	7.83 ± 1.51
Content needed	7	0–70	50.59 ± 15.18
Content received	7	0–70	48.45 ± 14.34
Delivery	13	0–130	108.09 ± 20.11
HL	34	0–34	28.27 ± 5.83
Basic knowledge and concepts	15	0–15	12.54 ± 2.84
Lifestyle and behaviour	12	0–12	10.93 ± 2.01
Basic skills	7	0–7	4.81 ± 2.13

Abbreviations: HL, health literacy; QDT, quality of discharge teaching; RHD, readiness for hospital discharge.

The QDT score was 0.9–10 (7.83 ± 1.51), and the sub‐dimension scores were 50.59 ± 15.18 (content needed), 48.45 ± 14.34 (content received) and 108.09 ± 20.11 (delivery) (Table 2).

The HL score was 0–34 (28.27 ± 5.83), and the sub‐dimension scores were 12.54 ± 2.84 (basic knowledge and concepts), 10.93 ± 2.01 (lifestyle and behaviour) and 4.81 ± 2.13 (basic skills) (Table 2).

### 3.4 Correlation Between RHD and the Study Variables

The total RHD score exhibited a significant positive correlation with the total QDT score (*r* = 0.387, *p* < 0.001), total HL score (*r* = 0.265, *p* < 0.001) and BMI (*r* = 0.125, *p* < 0.05). Furthermore, the total RHD score was significantly positively correlated with the ‘content received’ (*r* = 0.306, *p* < 0.001), ‘delivery’ (*r* = 0.396, *p* < 0.001), ‘basic knowledge and concepts’ (*r* = 0.145, *p* < 0.05), ‘lifestyle and behavior’ (*r* = 0.249, *p* < 0.001) and ‘basic skills’ (*r* = 0.277, *p* < 0.001) dimensions (Table 3).

**TABLE 3:** Correlation between RHD and age, BMI, QDT and HL (*n* = 308).

	RHD	Personal status	Knowledge	Coping ability	Expected social support
QDT	0.387^a^	0.309^a^	0.353^a^	0.363^a^	0.315^a^
Content needed	0.046	0.066	0.045	0.049	0.036
Content received	0.306^a^	0.217^a^	0.311^a^	0.277^a^	0.182^a^
Delivery	0.396^a^	0.337^a^	0.332^a^	0.390^a^	0.378^a^
HL	0.265^a^	0.125^b^	0.291^a^	0.301^a^	0.116^b^
Basic knowledge and concepts	0.145^b^	0.060	0.133^b^	0.199^a^	0.084
Lifestyle and behaviour	0.249^a^	0.125^b^	0.265^a^	0.269^a^	0.169^a^
Basic skills	0.277^a^	0.138^b^	0.327^a^	0.303^a^	0.078
Age	0.043	0.018	0.072	0.063	−0.016
BMI	0.125^b^	0.129^b^	0.133^b^	0.086	0.048

Abbreviations: HL, health literacy; QDT, quality of discharge teaching; RHD, readiness for hospital discharge.

^a^ The correlation at 0.01 level (two‐sided test) is statistically significant.

^b^ The correlation at 0.05 level (two‐sided test) is statistically significant.

### 3.5 Multiple Linear Regression Analysis of the Factors Affecting Maternal RHD

Stepwise multiple linear regression analysis was applied using the total RHD score as the dependent variable and factors with statistical significance in univariate analysis (*p* < 0.05) and those significantly correlated with RHD in the correlation analysis (*p* < 0.05) as independent variables. To assess multicollinearity among the independent variables, we calculated the variance inflation factor (VIF), with a threshold greater than 4 indicating potential collinearity issues.

The results showed that the ‘delivery’ dimension in the OB‐QDTS and the ‘lifestyle and behavior’ and ‘basic skills’ dimensions in the PMHLS were the primary factors affecting maternal RHD (adjusted *R* ^2^ = 0.221, *F* = 30.107, *p* < 0.001). The VIF of the independent variable was 1.002–1.283, indicating no multicollinearity problem among the variables (Table 4).

**TABLE 4:** Results of the Multiple Linear Regression Analysis (Stepwise) (*n* = 308).

Independent	*B*	SE	*β*	*t*	*p*	*VIF*
Constant	61.493	8.724	—	7.049	< 0.001	—
Lifestyle and behaviour	1.492	0.641	0.133	2.327	0.021	1.281
Basic skills	2.343	0.604	0.221	3.881	0.000	1.283
Delivery	0.399	0.057	0.355	7.045	0.000	1.002

*Note:* *F* = 30.107, *p* < 0.001, *R* = 0.479, *R* ^2^ = 0.229, *R* ^2^_adj_ = 0.221.

## 4 Discussion

In mainland China, where nearly 99.9% of women give birth in hospitals (Qiao et al. 2021), healthcare providers are required to provide puerperal healthcare for mothers and newborns (Ministry of Health of the People's Republic of China 2025). Exploring maternal RHD and its associated factors can help healthcare professionals prepare mothers for hospital discharge and the puerperal period more effectively.

In this study, the effective response rate was 86.27%, which was lower than that reported in Xia and Shi's (2024) study (95.9%) and Dandan et al.'s (2018) study (95.1%), probably because low‐quality questionnaires were not recognised on the spot during data collection. The reduction in sample size reduced the statistical power, increased the risk of false negatives, and may have rendered significant differences insignificant. In future research, participants with a low degree of willingness to complete questionnaires should be excluded. If all options in the questionnaire are the same or run in a loop, the individuals concerned should be verified and instructed on how to complete the questionnaire.

The overall maternal RHD score in this study was 132.17 ± 22.59, which was lower than the findings reported by Malagon‐Maldonado et al. (2017), Xia et al. (2023) and Yanıkkerem et al. (2018). Several factors may account for this discrepancy. First, the postpartum hospitalisation of the mothers in this study was short (1–3 days), during which they were physiologically and psychologically in the initial stage of postpartum recovery. The mothers experienced the process of delivery, the transition to motherhood, caring for the newborn, and discharge in this short time, which may have affected their ‘personal status’ and ‘coping ability’. Second, the mothers were likely to have been too busy taking care of their newborns and handling the discharge process after delivery to spend time and energy obtaining adequate discharge education, which may have resulted in insufficient knowledge and skills in self‐care and newborn care and reduced ‘coping ability’ (Xia et al. 2023). Third, to mitigate the risk of neonatal and puerperal infections, the participants in this study were permitted only one accompanying individual during hospitalisation. This restriction may have had adverse effects on their perceived ‘social support’. Additionally, the mothers were accommodated in double‐ or triple‐occupancy rooms, which could potentially have compromised sleep quality and affected their ‘personal status.’ Therefore, to enhance maternal preparedness, it is advisable to implement discharge health education prior to delivery, allowing sufficient time for mothers to assimilate the necessary information. Moreover, online health education videos could further facilitate mothers' access to discharge information, improving their ‘knowledge’ and ‘coping ability’ (Gün Kakaşçı and Durmaz 2022). Furthermore, adopting single‐room maternity care is recommended, in which each mother is provided with a private room. This arrangement would offer the opportunity for more comprehensive care by family and friends and significantly enhance sleep quality, improving ‘social support’ and RHD (Hall et al. 2023).

The findings of this study indicate that QDT significantly influenced maternal RHD, with the ‘delivery’ dimension identified as the most impactful factor. This finding highlighted the skills and effectiveness of the guidance provided by nursing staff, with the higher the score, the better the maternal RHD (*p* < 0.001). This finding was consistent with the results reported in Malagon‐Maldonado et al. (2017) and Xia et al. (2023). In the ‘delivery’ dimension, the item inquiring about maternal confidence in managing emergencies received the lowest score, which suggested a deficiency in maternal emergency coping skills and knowledge. Therefore, healthcare professionals should enhance health education and training for mothers, focusing on emergencies such as common postpartum complications, neonatal foreign body aspiration and neonatal asphyxia. Furthermore, the study revealed that the health information imparted to mothers before discharge did not adequately meet their needs. This inadequacy may stem from the limited duration of hospital stay and the substantial workload faced by healthcare professionals, which restricts the time available for comprehensive health guidance. Additionally, the absence of standardised health education in clinical practice may result in the omission of critical content during the educational process. To address these challenges, healthcare administrators should prioritise the allocation of an adequate workforce, optimise the scheduling of healthcare staff for discharge health guidance, and establish uniform, standardised health education protocols. These measures are essential to fully meet the needs of mothers and enhance the overall effectiveness of QDT.

The study highlights the critical role of HL in maternal RHD. Specifically, ‘lifestyle and behavior’ and ‘basic skills’ emerged as key determinants of maternal RHD. The statistical analysis revealed that higher scores within these dimensions corresponded to improved maternal RHD (*p* < 0.001), corroborating conclusions drawn in previous research (Waldron 2017; Lin et al. 2024). Within the two dimensions, particular focus is required on the three items exhibiting the lowest average scores: practices related to breast hygiene during breastfeeding, newborn bathing and newborn swimming. Inadequate breast washing practices may facilitate bacterial proliferation, potentially leading to the development of mastitis. The existing literature indicates a correlation between subclinical mastitis and slowed infant growth, which could have adverse effects, including being underweight, stunting and low head circumference in infants (Wren‐Atilola et al. 2019). To mitigate these risks, healthcare professionals should provide mothers with comprehensive guidance on the correct methods and recommended frequency of breast hygiene. Such education is beneficial for sustaining optimal breast health and minimising the incidence of mastitis, promoting healthier growth trajectories in infants. Furthermore, the advantages of bathing and swimming for newborns need to be emphasised, as these practices can enhance infants' gross and fine motor skills, visual‐motor perception, cognitive flexibility and response selection accuracy (Santos et al. 2023). Finally, healthcare providers should instruct mothers on the appropriate techniques for bathing and swimming while ensuring the safety of the newborns.

Nevertheless, the effect of maternal age on RHD in this study was not statistically significant, which differs from the results of several prior investigations. Xia and Shi (2024) identified a trend of increasing RHD associated with advancing maternal age. Conversely, Ran et al. (2022) reported a reduction in RHD with increasing age. These discrepancies may be attributed to variances in the age cohorts of the mothers included in the analyses. In this study, the mothers' ages ranged from 20 to 41 years, with a mean age of 31.15 ± 4.14 years, and the majority (76.3%) fell within the 26–35 year age bracket. Differences in cognitive abilities, physical health, skills and life experiences among mothers in this relatively narrow age range were minimal, which may account for the lack of a significant effect of maternal age on RHD observed in this study. Additionally, Xia and Shi (2024) study used a self‐made research questionnaire developed by the team, and its effectiveness and applicability need to be verified through further research. Ran et al. (2022) study only included women who had undergone caesarean section for the first time, whereas the participants in this study included women who had undergone caesarean section and vaginal delivery. These differences may explain the inconsistent results. To better elucidate the influence of maternal age on RHD, future research should consider broadening the age range, unifying research tools and populations and increasing the sample size.

Finally, this study found that maternal education level did not have a statistically significant effect on RHD. That result differed from previous research findings that reported that mothers with higher education levels tended to have better access to knowledge, greater enthusiasm for learning, and a more comprehensive understanding of postnatal rehabilitation and infant care that allowed them to take better care of themselves and parent their children more effectively, resulting in higher discharge preparedness (Xia and Shi 2024; Dandan et al. 2018). In this study, the differences in RHD among mothers with different education levels were not apparent, probably because of differences in regional medical and health policies. The mothers in Xia and Shi (2024) study had already received postpartum health education in prenatal courses, and the differences in knowledge mastery among mothers with different levels of education became apparent over time. In this study, postpartum health education was conducted during the hospitalisation period of the mothers after delivery (usually 1–3 days). During this limited timeframe, they were primarily focused on caring for their newborns and completing discharge procedures and did not fully engage with the discharge education offered by healthcare professionals. Thus, there was no difference in RHD among mothers with different education levels. To address this issue, healthcare professionals could incorporate topics such as puerperal care, postpartum rehabilitation, newborn bathing and breastfeeding into the prenatal curriculum and encourage mothers to acquire the necessary knowledge and skills independently before delivery. This process would establish a strong foundation for acquiring rehabilitation and parenting skills after birth and effectively enhance RHD.

## 5 Limitations

First, this study was conducted at a single tertiary hospital, which limits the generalisability of the findings. Future research should consider large‐scale, multicentre surveys that encompass diverse regions and healthcare institutions to enhance the applicability of the results. Second, this study predominantly examined maternal perceptions of RHD, neglecting the perspectives of other stakeholders. Incorporating insights from family members and healthcare professionals could provide a more comprehensive understanding of maternal experiences related to RHD. Finally, the admission of neonates to the neonatal intensive care unit may have resulted in several mothers being discharged without their infants. This circumstance may have influenced maternal RHD and should be considered in future studies.

## 6 Conclusion and Implications

This study found that maternal RHD was independently associated with the ‘delivery’ dimension of the OB‐QDTS, as well as the ‘lifestyle and behavior’ and ‘basic skills’ dimensions of the PMHLS. Medical staff should introduce postpartum discharge education into the curriculum for pregnant women during prenatal care. Additionally, standardised discharge health guidance should be developed to enhance maternal RHD by improving the quality of discharge education and HL.

## Funding

This work was supported by the Seventh Affiliated Hospital of Sun Yat‐Sen University (Grant No. ZSQYHLKYJJ202302).

## Conflicts of Interest

The authors declare no conflicts of interest.

## Data Availability

The data that support the findings of this study are available on request from the corresponding author.
